# Doniach Lattice Gas on Bipartite Lattices in the Mean-Field Approximation

**DOI:** 10.1021/acs.langmuir.4c04225

**Published:** 2025-05-09

**Authors:** C. P. B. Vignoto, M. N. Tamashiro

**Affiliations:** 28132 Universidade Estadual de Campinas (UNICAMP) Instituto de Física Gleb Wataghin, Rua Sérgio Buarque de Holanda, 777, Cidade Universitária, Campinas SP, 13083-859, Brazil

## Abstract

The Doniach lattice gas (DLG) consists of a statistical model that can be mapped into a spin-1 Ising model with highly degenerate single-site states and the inclusion—using the nomenclature of the analogous magnetic model—of dipole–quadrupole interactions, besides the usual dipole–dipole, Zeeman-effect and crystal–field interactions. Its formulation was motivated aiming at the study of phase transitions in supramolecular structures of zwitterionic phospholipids, in particular, allowing an alternative description of density fluctuations in the system, already included in a certain class of lattice models (Nagle, J. F. *J. Chem. Phys.* **1973**, *58*, 252; Nagle, J. F. *J. Chem. Phys.* **1975**, *63*, 1255), but not considered in previous proposals of Ising-type models (Doniach, S. *J. Chem. Phys.* **1978**, *68*, 4912). In this work, we investigate the DLG model, considering the division of the system into two interpenetrating sublattices, under the framework of the mean-field approximation. This analysis of the model on bipartite lattices allowed the investigation of staggered phases, which were overlooked in the first analysis of the model in the mean-field approach (Guidi, H. S.; Henriques, V. B. *Phys. Rev. E* **2014**, *90*, 052705), precisely because it was only assumed the presence of uniform phases, i.e., without splitting the system into two distinct sublattices. However, such staggered phases were observed for this model in the pair approximation on bipartite lattices (de Oliveira, F. O.; Tamashiro, M. N. *Phys. Rev. E* **2019**, *99*, 012147; de Oliveira, F. O.; Tamashiro, M. N. *Langmuir* **2019**, *35*, 3848). Throughout the work, in addition to the staggered phase, we also observed intermediate topologies of representative phase diagrams (μ̅/*z*, *t*/*z*), which explain the development of the main topologies as we change the parameters ( l̅ , *k̅*) associated with the effective Hamiltonian interactions. Finally, we perform a parameter fitting between theoretical results and isothermal compression experimental data for the phospholipid 1,2-dimyristoyl-*sn*-glycero-3-phosphocholine (DMPC), allowing also a comparison between the fittings obtained using the mean-field and the pair approximations.

## Graphical abstract

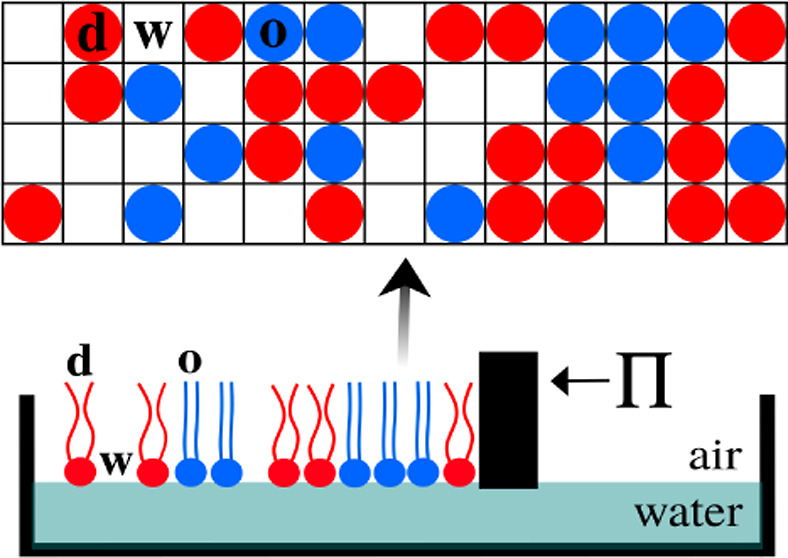

## Introduction

The cell membranes of almost all living organisms are essentially formed by amphiphilic molecules, especially phospholipids, that are self-assembled in the form of semifluid bilayers.^1^ The cell-membrane structure, formed immersed in an aqueous solutions, has other biological macromolecules aggregated, such as proteins and cholesterol, in order to keep its biological function of compartmentalization. The two fatty-acid (hydrocarbonic) chains or tails, that usually form the phospholipids present in bilayers, have a stronger interaction between themselves than with the surrounding water molecules, having an effective repulsive tail−water interaction, which keeps the tails inside of the membrane. On the other hand, the polar heads have an effective attractive interaction with water, in order to organize themselves at the extracellular and intracellular interfaces of the bilayer.^2^ The so-called Langmuir films or monolayers are an interesting experimental system that mimetizes the cell membranes.^3,4^ These are composed by a single amphiphile layer present at the air–water interface, with the hydrocarbon chains pointed toward the air and the polar heads resting on water. In this structure, the monolayer tends to spread over the surface at certain area extensions, being possible to control it through an external lateral pressure, unlike bilayers, that are tension-free, stabilized by the hydrophobic effect of the apolar chains.

Both systems can undergo several phase transitions, but although aqueous solutions containing bilayers can be directly investigated, the Langmuir monolayers represent a rather more accessible and controllable experimental setup, since we have an associated lateral pressure. While phase transitions in bilayers occur at a fixed temperature for each type of phospholipid, in monolayers they can occur by variations in the available area or by changing the temperature while keeping this area constant. In both systems, the main transition has an order–disorder type, where we have a medium-density to a high-density phase transition. For lipid membranes this transition is called main gel–fluid transition, while for monolayers it is traditionally known as the liquid condensed−liquid expanded (LC-LE) transition. Another phase transition commonly found in monolayers is the gas–liquid expanded (G-LE) transition, that is a low-density to a medium-density phase transition.^3,4^ After years of discussion about the nature of these two phase transitions in monolayers,^5–8^ nowadays it is recognized that both are discontinuous (first-order) transitions, displaying thus an associated latent heat.^3,4^ A continuous (second-order) transition, liquid condensed−​solid crystalline (LC-SC) transition, may be observed by increased compression, but will not be considered in this work. The monolayer eventually expands to three dimensions and may even collapse by taking higher surface pressures.^9^ From the gel phase, as we increase the temperature, some lipid bilayers assume a still not well-understood so-called ripple phase, which has a periodically undulated structure. The bilayer transition between the gel and the ripple phases is called pretransition, since it occurs at lower temperatures than the main gel–fluid transition, correlated to the monolayer LC-LE transition. Despite being widely presented in the literature,^10–15^ the physical understanding of the ripple phase still represents a major challenge.^16–24^

From a theoretical point of view, there are several proposals for modeling phase transitions in zwitterionic phospholipids,^25,26^ for which the hydrophilic headgroup of the molecules, although polar, has a null net charge. Nagle^27^ presented a successful pioneering proposal, whose approach describes the entropy of the lipid chains and can be exactly solved for the case of tails of infinite length by mapping the problem on a dimer model; density fluctuations were included in the model in subsequent work.^28^ Later, Doniach^29^ proposed a simplified approach to the lipid problem by considering a mapping into the familiar two-dimensional spin-1/2 ferromagnetic Ising model.^30–32^ The Doniach model^29^ consists of a two-dimensional lattice model with two possible states and interactions between nearest-neighbors lipids. The lipid molecules can be in two states: ordered (gel-like) state (o), with extended and laterally compact configuration, or a disordered (liquid-like) state (d), which has a high degeneracy ω ≫ 1 attributed to the rotamers of the lipid chains in this state. As for the two-dimensional spin-1/2 ferromagnetic Ising model, we have an order–disorder first-order phase transition, which occurs between the lipid phases mainly with ordered-chains (LC) and disordered-chains (LE) states, respectively. Although the Doniach model presents a behavior similar to that observed in monolayer experiments, it still has several limitations. The model does not describe the lipid-density fluctuations, since the development of the model fixed *ad hoc* distinct areas per molecule for the two lipid states. Even though experimentally the area per lipid *a*_0_ in the ordered state is almost constant, the average area per lipid in the disordered state tends to vary greatly with temperature. Also, the model does not allow the description of the G-LE transition.

The Doniach lattice gas (DLG) model^33–35^ was then proposed as an extension of the Doniach model^29^ in order to overcome its limitations. To allow lipid-density fluctuations, the DLG model introduces a new vacant state (w)—representing lattice sites filled by water molecules—in addition to the two lipid states ordered (o) and disordered (d). The DLG model can be written in terms of spin-1 variables, where the state *s*_*i*_ = +1 represents the ordered state, *s_i_* = 0 the vacant state and *s*_*i*_ = −1 the disordered state of lipids, which is associated with a high degeneracy ω ≫ 1 that represents an average over all possible configurations (twisting) that the hydrocarbon chains can assume. The DLG model is described on a regular two-dimensional lattice that has a fixed area *A* = *Na*_0_, with the sites occupying an elementary area determined by the lattice parameter $∼\sqrt{a_{0}}$ . The effective model Hamiltonian can be written,^33–35^ in the grand-canonical ensemble, in terms of spin-1 variables, with the single-site states (d, o, w) at lattice site *i* = 1, ..., *N* represented by variables *s*_*i*_ = −1, +1, 0, respectively, and (*i*, *j*) represents a sum over its *z* nearest-neighbor sites

1 $$-H=E_{0}+J\sum_{\left(i,j\right)}s_{i}s_{j}+K\sum_{\left(i,j\right)}s_{i}^{2}s_{j}^{2}+\frac{1}{2}L\sum_{\left(i,j\right)}s_{i}s_{j}\left(s_{i}+s_{j}\right)+H\sum_{i}s_{i}+\mu_{eff}\sum_{i}s_{i}^{2}$$

It should be remarked that, due to the independent local degeneracies ω of each lipid in its disordered state, the system microstate {*s*_*i*_} acquires a global degeneracy factor, Ω­({*s_i_*}) = ω^∑_*i*_ *s_i_* (*s_i_* − 1)/2^, that has to be considered in the evaluation of the grand-canonical partition function Ξ ≡ $\sum_{\left\{s_{i}\right\}}\Omega \left(\{s_{i}\}\right)\;e^{-\beta H\left(\{s_{i}\}\right)}$ , with β ≡ (*k*_B_ *T*)^−1^. The reference energy *E*_0_ and the Hamiltonian parameters (*J*, *K*, *L*, *H*, μ_eff_) are linear combinations of the single-site intramolecular energies −ϵ_*x*_ and of the short-ranged attractive pairwise interactions −ϵ_*xy*_ between the nearest-neighbors sites, with *x* ∈ (d, o, w), *y* ∈ (d, o, w), and are given by^33–35^

E0=N(12zϵww+ϵw+μ w)(2)J=14(ϵdd+ϵoo−2ϵod)(3)K=14[ϵdd+ϵoo+2ϵod−4(ϵwd+ϵwo−ϵww)](4)L=12(ϵoo−ϵdd+2ϵwd−2ϵwo)(5)H=12[ϵo−ϵd+z(ϵwo−ϵwd)](6)μeff=μlip−μ w−z2(2ϵww−ϵwo−ϵwd)⁣−12(2ϵw−ϵo−ϵd)(7) 2

where μ_lip_ and μ_w_ are the chemical potentials of the lipid and the water, respectively.

This model represents an extension of the Blume–Emery–Griffiths (BEG) model^36^ with a dipole–quadrupole interaction (cubic terms), originally proposed in the context of simple fluids, binary and ternary mixtures.^37–40^ It is worth mentioning that a few spin-1 models presented in the literature to describe monolayer systems predict second-order (continuous) transitions for the LC-LE and G-LE transitions,^41–46^ in disagreement with the current hypothesis that they are, in fact, first-order (discontinuous) transitions.^5–8^

In the first proposal of the DLG model,^33^ in order to obtain some phase diagrams, the authors performed a general mean-field approximation (MFA) analysis and some Monte Carlo simulations for some cases of interest. Especially, the investigated parameters of the model covered only the critical point of the G-LE first-order phase transition, leaving out the critical point of the LC-LE phase transition, also of discontinuous (first-order) type. In a recent work, the DLG model was analyzed at the pair-approximation level^34,35^ (Bethe–Peierls approximation, BPA), where a Bethe−Gujrati^47^ lattice scheme with two sublattices was used, which provides the self-consistent grand potential directly at the pair-approximation level, allowing the detection of possible staggered (Stg) or modulated phases. In this approach, a wider range of parameter sets of the DLG model was investigated, obtaining some different topologies for the phase diagrams, including the existence of a Stg phase that was overlooked in the previous MFA analysis.^33^ Also, in this work,^34,35^ an explicit comparison of theoretical predictions at the pair-approximation level was made with data from isothermal compression experiments.^48^

The purpose of the current work is then to reanalyze the DLG model,^33–35^ again under the framework of the MFA, but now considering, in the section “Theoretical Section: DLG Model in the Mean-Field Approximation”, the division of the system into two interpenetrating sublattices. Our results are presented in the “Results and Discussion”. The first subsection, “Theoretical Phase Diagrams: Staggered Phase and New Phase-Diagram Topologies”, displays, in particular, the novel phase-diagram topologies and confirms the occurrence of the Stg phase for certain parameter sets, that was overlooked in the previous MFA analysis.^33^ In the second subsection, “Theory Versus Experiments”, we perform a parameter fitting between theoretical results and isothermal compression experimental data for the phospholipid 1,2-dimyristoyl-*sn*-glycero-3-phosphocholine (DMPC), allowing also a comparison between the fittings obtained using MFA and BPA. Some final comments are presented in the “Conclusions” section. Appendices I to IV provide details on some specific technical issues to locate spinodal and critical lines, and multicritical points.

## Theoretical Section: DLG Model in the Mean-Field Approximation

An approximate solution for the DLG model can be found by using a MFA. Instead of solving the short-ranged version of the model Hamiltonian, in which a particular site *i* interacts only with its *z* nearest neighbors *j*, we consider a system in which all *N* spins interact equally with each other, regardless of their relative position on the lattice. Furthermore, an approach inspired by the Bragg−Williams approximation^49,50^ is applied, by assuming a vanishing connected correlation function, ∑_(*i,j*)_(*s_i_* − ⟨*s_i_* ⟩)­(*s_j_* − ⟨*s_j_* ⟩) = 0, where ⟨···⟩ denotes thermal averages. Therefore, by considering the translational invariance of the order parameters for a uniform system, *m* = ⟨*s*_*i*_⟩, *q* = ⟨*s*_*i*_^2^⟩, the three nearest-neighbor interaction terms of the model Hamiltonian ([Disp-formula eq1]) can be replaced by decoupled single-site sums

8 $$J\sum_{\left(i,j\right)}s_{i}s_{j}\to \frac{1}{2N}Jz\sum_{i,j}\left(s_{i}\left\langle s_{j}\right\rangle +\left\langle s_{i}\right\rangle s_{j}-\left\langle s_{i}\right\rangle \left\langle s_{j}\right\rangle \right)=Jmz\sum_{i}\left(s_{i}-\frac{1}{2}m\right)$$

9 $$K\sum_{\left(i,j\right)}s_{i}^{2}s_{j}^{2}\to \frac{1}{2N}Kz\sum_{i,j}\left(s_{i}^{2}\left\langle s_{j}^{2}\right\rangle +\left\langle s_{i}^{2}\right\rangle s_{j}^{2}-\left\langle s_{i}^{2}\right\rangle \left\langle s_{j}^{2}\right\rangle \right)=Kqz\sum_{i}\left(s_{i}^{2}-\frac{1}{2}q\right)$$

10 $$\frac{1}{2}L\sum_{\left(i,j\right)}s_{i}s_{j}\left(s_{i}+s_{j}\right)\to \frac{1}{2N}Lz\sum_{i,j}\left(s_{i}\left\langle s_{j}^{2}\right\rangle +\left\langle s_{i}\right\rangle s_{j}^{2}-\left\langle s_{i}\right\rangle \left\langle s_{j}^{2}\right\rangle \right)=Lz\sum_{i}\frac{1}{2}\left(qs_{i}+ms_{i}^{2}-mq\right)$$

and the effective Hamiltonian written in MFA is given by

11 $$-\beta H_{MFA}\equiv -\beta H+\ln\Omega \left(\{s_{i}\}\right)=-\frac{1}{2}N\left(jm^{2}+kq^{2}+lmq\right)+(h-\frac{1}{2}\ln\omega +jm+\frac{1}{2}lq)\sum_{i=1}^{N}s_{i}+\left(\mu +\frac{1}{2}\ln\omega +kq+\frac{1}{2}lm\right)\sum_{i=1}^{N}s_{i}^{2}$$

in terms of the dimensionless parameters

12 $$j\equiv \beta Jz,\;k\equiv \beta Kz,\;l\equiv \beta Lz,\;h\equiv \beta H,\;\mu \equiv \beta \mu_{\mathrm{eff}}$$

The associated MFA grand-canonical partition function, Ξ­(*T*, *A* = *Na*_0_, *h*, μ) ≡ e^–βΨ^ = e^–*N*ψ^, and the functional of the dimensionless grand-canonical potential density per site ψ­(*h*, μ) ≡ βΨ/*N* can be easily obtained due to the factorization of Ξ and can be written as

13 $$\Xi \left(T,Na_{0},h,\mu \right)\equiv \sum_{\left\{s_{i}\right\}}\Omega \left(\{s_{i}\}\right)\;e^{-\beta H\left(\{s_{i}\}\right)}\equiv \sum_{\left\{s_{i}\right\}}e^{-\beta H_{\;MFA}\left(\{s_{i}\}\right)}=\exp\left[-\frac{1}{2}N\left(jm^{2}+kq^{2}+lmq\right)\right]\sum_{\left\{s_{i}\right\}}\prod_{i=1}^{N}\exp\left(\eta s_{i}+\theta s_{i}^{2}\right)=\exp\left[-\frac{1}{2}N\left(jm^{2}+kq^{2}+lmq\right)\right]{\left(1+2e^{\theta }\cosh\eta \right)}^{N}$$

14 ψ(h,μ)=12(jm2+kq2+lmq)−ln(1+2eθcoshη)(14)η≡h−12ln⁡ω+jm+12lq(15)θ≡μ+12lnω+kq+12lm(16)

Convenient partial derivatives of the grand-canonical potential density per site yield the equations of state that define the thermodynamic order parameters,

17 $$m\left(h,\mu \right)\equiv \left\langle s_{i}\right\rangle =\frac{1}{N}{\left(\frac{\partial \;\ln\Xi }{\partial h}\right)}_{\mu }=-{\left(\frac{\partial \psi }{\partial h}\right)}_{\mu }=\frac{2\;e^{\theta }\;\mathrm{senh}\;\eta }{1+2\;e^{\theta }\;\cosh\eta }$$

18 $$q\left(h,\mu \right)\equiv \left\langle s_{i}^{2}\right\rangle =\frac{1}{N}{\left(\frac{\partial \;\ln\Xi }{\partial \mu }\right)}_{h}=-{\left(\frac{\partial \psi }{\partial \mu }\right)}_{h}=\frac{2\;e^{\theta }\;\cosh\eta }{1+2{\;e}^{\theta }\;\cosh\eta }$$

which are consistent with the functional minimization of ψ with respect to (*m*, *q*), i.e., (∂ψ/∂*m*)_*q,h,μ*_ = (∂ψ/∂*q*)_*m,h,μ*_ = 0. This system of equations can be interpreted as a nonlinear mapping, whose limits of numerical stability of its solutions, associated with the corresponding Jacobian matrix, are obtained in Appendix I. Some algebraic manipulations allow us to obtain the conjugate thermodynamic fields (*h*, μ) in terms of the order parameters (*m*, *q*),

19 h(m,q)=12ln(q+mq−m)−jm−12lq+12ln⁡ω(19)μ(m,q)=12ln(q+m)+12ln(q−m)−ln[2(1−q)]⁣−kq−12lm−12ln⁡ω(20)

These expressions will be useful when analyzing the multicritical behavior of the system, for which the Helmholtz representation *f* = *f*(*m*, *q*) is more suitable. The multicritical conditions involving only uniform phases are given in Appendix II.

Different choices of Hamiltonian parameter sets can produce different phase diagrams. The original DLG paper^33^ presents some (μ, *t* ≡ 1/*j*) phase diagrams in MFA—see, e.g., Figures 4, 7, and 12 of ref 33—which display a G-LE first-order transition line ending at a critical point. In subsequent work of the DLG model investigated at the pair approximation,^34^ the calculations were performed recursively on the Cayley tree, whose approximate solutions at the center of the tree are equivalent to those obtained by the traditional Bethe−Peierls approximation (BPA) on a regular lattice. Within this improved approximation, the authors displayed typical phase-diagram topologies for the DLG model under BPA—see, e.g., Figure 2 of ref 34—which were consistent with an asymptotic analysis, presented in Appendix of ref 33 for MFA, and extended to BPA by a correction factor ϕ­(*z*) in Appendix C of ref 34. Also, in this BPA work, it was detected the existence of Stg phases, overlooked in ref 33 at the MFA.

Therefore, in order to analyze the possibility of occurrence of Stg phases at the MFA, we need to reformulate the problem on a bipartite lattice, splitting the system into two interpenetrating sublattices *a* and *b*. A spin in a given sublattice interacts equally with all the spins in the other sublattice, that is, a spin located at a site *i* in the sublattice *a* interacts with all the spins *j* in the sublattice *b*, and vice versa. It is convenient to formally introduce distinct conjugated fields for each sublattice, ***h*** ≡ (*h*_*a*_, *h*_*b*_) and **μ** ≡ (μ_*a*_, μ_*b*_), and their associated order parameters, ***m*** ≡ (*m*_*a*_, *m*_*b*_) and ***q*** ≡ (*q*_*a*_, *q*_*b*_). Analogously to the system with a uniform lattice, we can write the associated MFA grand-canonical partition function, Ξ­(*T*, *Na*_0_, ***h***, **μ**), and the functional of the dimensionless grand-canonical potential density per site, ψ­(***h***, **μ**):

21 $$\Xi \left(T,Na_{0},h,\mu \right)=\exp\left[-\frac{N}{2}\left(jm_{a}m_{b}+kq_{a}q_{b}+\frac{l}{2}m_{a}q_{b}+\frac{l}{2}m_{b}q_{a}\right)\right]\times {\left(1+2\;e^{\theta_{a}}\;\cosh\eta_{a}\right)}^{N/2}{\left(1+2\;e^{\theta_{b}}\;\cosh\eta_{b}\right)}^{N/2}$$

22 $$\psi \left(h,\mu \right)=\frac{1}{2}\left(jm_{a}m_{b}+kq_{a}q_{b}\right)+\frac{1}{4}l\left(m_{a}q_{b}+m_{b}q_{a}\right)-\frac{1}{2}\ln\left(1+2\;e^{\theta_{a}}\;\cosh\eta_{a}\right)-\frac{1}{2}\ln\left(1+2\;e^{\theta_{b}}\;\cosh\eta_{b}\right)$$

23 ηa≡ha−12ln⁡ω+jmb+12lqb(23)θa≡μ a+12ln⁡ω+kqb+12lmb(24)ηb≡hb−12ln⁡ω+jma+12lqa(25)θb≡μb+12ln⁡ω+kqa+12lma(26)

As previously obtained for a uniform system, the equations of state for each sublattice are given by appropriate partial derivatives of the dimensionless grand-canonical potential density, eq 22:

27 ma(h,μ)≡⟨si⟩a=−2(∂ψ∂ha)hb,μ=2eθasenhηa1+2eθacosh⁡ηa(27)qa(h,μ)≡⟨si2⟩a=−2(∂ψ∂μ a)h,μb=2eθacosh⁡ηa1+2eθacoshηa(28)mb(h,μ)≡⟨si⟩b=−2(∂ψ∂hb)ha,μ=2eθbsenhηb1+2eθbcosh⁡ηb(29)qb(h,μ)≡⟨si2⟩b=−2(∂ψ∂μb)h,μ a=2eθbcosh⁡ηb1+2eθbcosh⁡ηb(30)

which are consistent with the functional minimization of ψ with respect to (***m***, ***q***). The multicritical conditions involving Stg phases on bipartite lattices are given in Appendix III.

According to the definition of the dimensionless parameters, eq 12, at the MFA level, all spin–spin interaction parameters scale to the coordination number *z*. Therefore, to present the numerical results in a general way, regardless of the lattice coordination *z*, we redefine the set of dimensionless parameters ([Disp-formula eq12]) by dimensionless parameters scaled to the bilinear coupling *J*

31 tz≡1βJz=1j,⁣k̅≡KJ=kj,⁣l̅≡LJ=lj,h̅z≡HJz=hj,⁣μ̅z≡μeffJz=μj(31)

## Results and Discussion

### Theoretical Phase Diagrams: Staggered Phase and New Phase-Diagram Topologies

Figure 2 of ref 34 displays six typical (μ̅/*z*, *t*/*z*) topologies of phase diagrams, which are reproduced in the top part of Figure 1. Note, especially, that there are two phase-diagram topologies, cases (e_0_) and (f_1_), with the presence of a Stg phase. In the original DLG paper,^33^ the range of parameters in which the Stg phase occurs was overlooked. Although the performance of the BPA is expected to be better than that of the MFA, we present the MFA results considering now the possibility of existence of Stg phases, making necessary to split the system into two interpenetrating sublattices. Thus, we confirm that the occurrence of the Stg phase is not due to the chosen improved BPA approximation, as it occurs already at the MFA level when a proper account of a bipartite lattice is considered.

**Figure 1:**
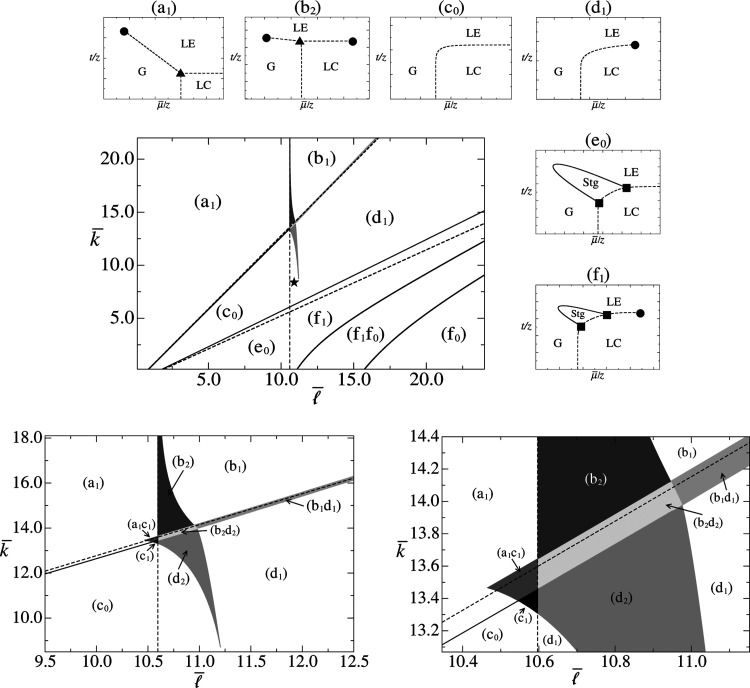
Reconfiguration of the ( l̅ , *k̅*) diagram after the determination of new topologies of the phase diagrams, obtained for parameters *h̅* = 0 and ω = 4 × 10^4^. The two bottom diagrams represent magnifications of the intermediate shaded subregions, to allow a better visualization. The dashed straight lines are associated with the asymptotic analysis proposed in refs 33 and 34—for MFA they are given by eqs 32−34 by taking ϕ­(*z*) = 1: ${\mathrm{l̅}}_{+}\left(\mathrm{k̅}\right)$ [(a_1_/c_0_), (b_1_/d_1_)]; ${\mathrm{l̅}}_{0}$ [(a_1_/b_1_), (c_0_/d_1_), (e_0_/f_1_)]; ${\mathrm{l̅}}_{-}\left(\mathrm{k̅}\right)$ [(c_0_/e_0_), (d_1_/f_1_)]—while the solid lines, as well as the edges of the shaded regions, represent the actual boundaries of each region, obtained numerically with the complete spin-1 DLG model. The star symbol (★) indicates the fitting parameters of the MFA theoretical results with experimental data of compression isotherms of DMPC, presented in Figure 4. Around the ( l̅ , *k̅*) diagram the six main typical (μ̅/*z*, *t*/*z*) topologies of phase diagrams—obtained previously in refs 33 and 34—are displayed: (a_1_), (b_2_), (c_0_), (d_1_), (e_0_) and (f_1_). For these typical phase diagrams, the dashed lines represent first-order (discontinuous) phase transitions between the standard (G, LE, LC) phases and between the Stg and LC phases. The solid lines represent second-order (continuous) phase transitions between the Stg and the (G, LE) phases. The special points are indicated by symbols: critical point (●), triple point (▲) and critical end point (■). The same representation for the thermodynamic phases, transition lines and symbols for the special points will be used in Figures 2 and 3, where the remaining (b_1_), (f_1_f_0_), (f_0_) and intermediate-regions topologies are portrayed in more detail.

Related to the critical behavior of the transitions, the asymptotic analysis proposed in the Appendix of ref 33 is based on mapping the DLG model into equivalent spin-1/2 Ising models in three distinct limits, corresponding to the first-order transitions G-LE, G-LC and LE-LC. In Appendix C of ref 34, the authors extended the asymptotic analysis originally done in MFA to BPA, in order to relate the critical conditions between the two approximations by a correction factor ϕ­(*z*) ≡ *t*_c_(*z*)/*t*_c_^MFA^(*z*) given by the ratio of the critical temperatures of the spin-1/2 ferromagnetic Ising model under the considered approximation (BPA) and MFA. In this way, critical conditions in MFA are recovered by taking the infinity-coordination limit ϕ­(*z*→*∞*) → 1. Through this asymptotic analysis, it is possible to produce a ( l̅ , *k̅*) diagram, as shown in Figure 7 of ref 34—updated and reproduced in Figure 1—partitioned by straight lines defined by the functions

32 l̅+(k̅)≡1+k̅1+4ϕ(z)ln⁡⁡ω−2h̅z[1+14ϕ(z)ln⁡ω]−1(32)l̅0≡ϕ(z)ln⁡ω−2h̅z(33)l̅−(k̅)≡1+k̅1−4ϕ(z)ln⁡ω−2h̅z[1−14ϕ(z)ln⁡ω]−1(34)

which maps and summarizes the regions associated with different topologies of the (μ̅/*z*, *t*/*z*) phase diagrams. Figure 1 displays the updated ( l̅ , *k̅*) diagram, since in our further investigation, other possible topologies for (μ̅/*z*, *t*/*z*) phase diagrams were obtained, in addition to those already found in refs 33 and 34 . To improve visualization, in the bottom part of Figure 1 there are enlargements of the intermediate shaded subregions, some of which are quite narrow. We still considered the parameter *h̅* = 0 and the degeneracy factor ω suitable to experimental values of the zwitterionic phospholipid DMPC, ω = 4 × 10^4^.^51^ However, all the investigated phase diagrams here correspond only to theoretical predictions. The old (a) to (f) regions in the ( l̅ , *k̅*) diagram of ref 34 were renamed by (a_1_), (b_2_), (c_0_), (d_1_), (e_0_) and (f_1_), as shown in Figure 1. The new subscripted-index nomenclature follows now the pattern of the number of critical points present in the typical phase diagram of each region. With the exception of the region (b_2_)—which corresponds to the subregion close to the boundary between the regions (a) and (b)—all the other regions correspond to the main subregion within a given region.

The new topologies for the μ̅/*z* × *t*/*z* phase diagrams are displayed in Figures 2 and 3, and the values of the pairs (*k̅*, l̅ ) used to exemplify each topology are listed in Table 1. Figure 2 shows the new cases referring to the shaded gray regions in Figure 1, which represent regions intermediate to the main old regions (a) to (d). In these cases we highlight the presence of a first-order phase transition between two distinct LE phases, LE1-LE2, except in the case (b_1_). By the analysis at the BPA level,^34^ it was initially proposed that in region (b) we would find only phase diagrams with topologies of the case (b_2_), but the emergence of diagrams of type (b_1_) for greater values of l̅ showed that the case (b_2_) only occurs near the region bordering the case (a_1_), while case (b_1_) dominates the rest of the old region (b). Depending on the choice of the parameters *k̅* and l̅ , we can change the topology of the diagrams, so that the different lines of first-order phase transitions can shrink, ceasing to be a physically stable phase transition, and becoming a numerically metastable transition (not shown in Figures 2 and 3). On the other hand, as a first-order phase-transition line shrinks, another tends to lengthen and may take its place, as in region (d), which is composed of region (d_1_) separated in two subregions by the intermediate region (d_2_). The region (d_1_) to the left of the region (d_2_) presents the topology of a LE-LC critical point associated with its first-order transition line, with this critical point arising from the shrinkage of the LE-LC transition line of the region (c_0_), which has no LE-LC critical point. As we increase the parameter l̅ and enter into the region (d_2_), the first-order transition LE1-LE2 between two distinct LE phases also appears, leading to the topology given in the fourth diagram of Figure 2. Proceeding with increasing l̅ , the first-order transition line LE1-LE2 grows, while the first-order transition line LE2-LC decreases, and when moving to the rightmost region (d_1_) again, the first-order transition LE1-LE2 gives rise to the first-order transition LE-LC.

**Figure 2:**
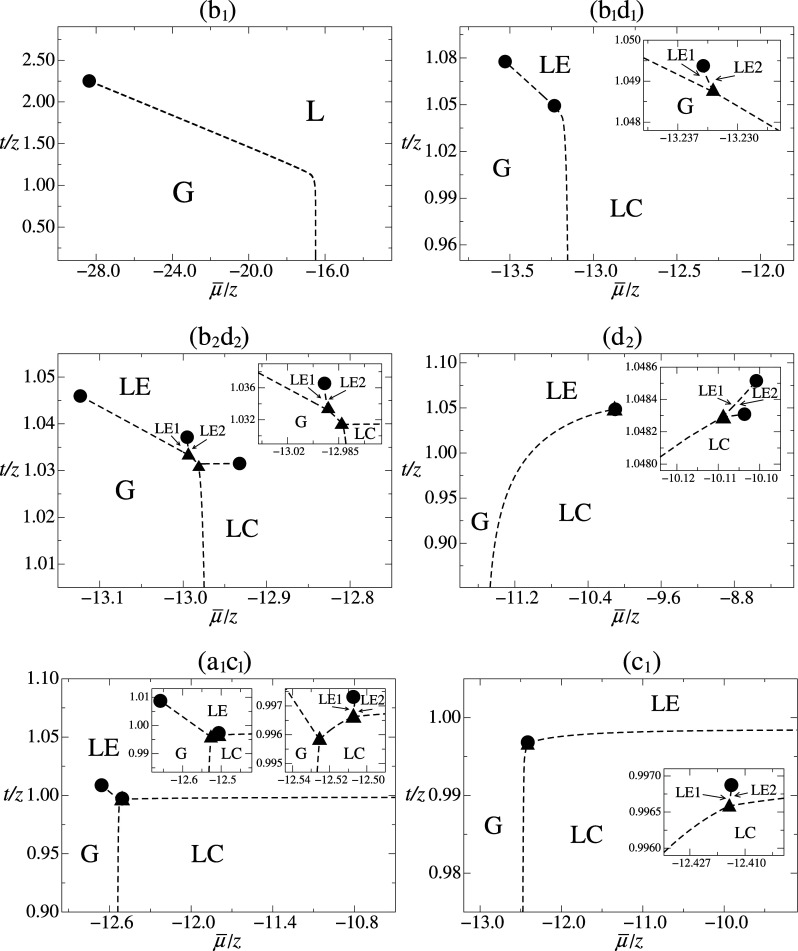
Dimensionless temperature *t*/*z* versus μ̅/*z* typical phase diagrams obtained for the DLG model under MFA for *h̅* = 0 and ω = 4 × 10^4^ for the intermediate shaded subregions presented in Figure 1. The pair values (k̅,l̅) corresponding to each region are given in Table 1. Besides the standard (G, LE, LC) phases, there are LE1 and LE2 phases that represent two distinct LE phases, indicated by the arrows. The same representation introduced in Figure 1 for the thermodynamic phases, transition lines and symbols for the special points have been used here. The insets represent magnifications of the regions where the two LE1 and LE2 phases coexist, with the associated first-order transition line ending at a critical point.

**Figure 3:**
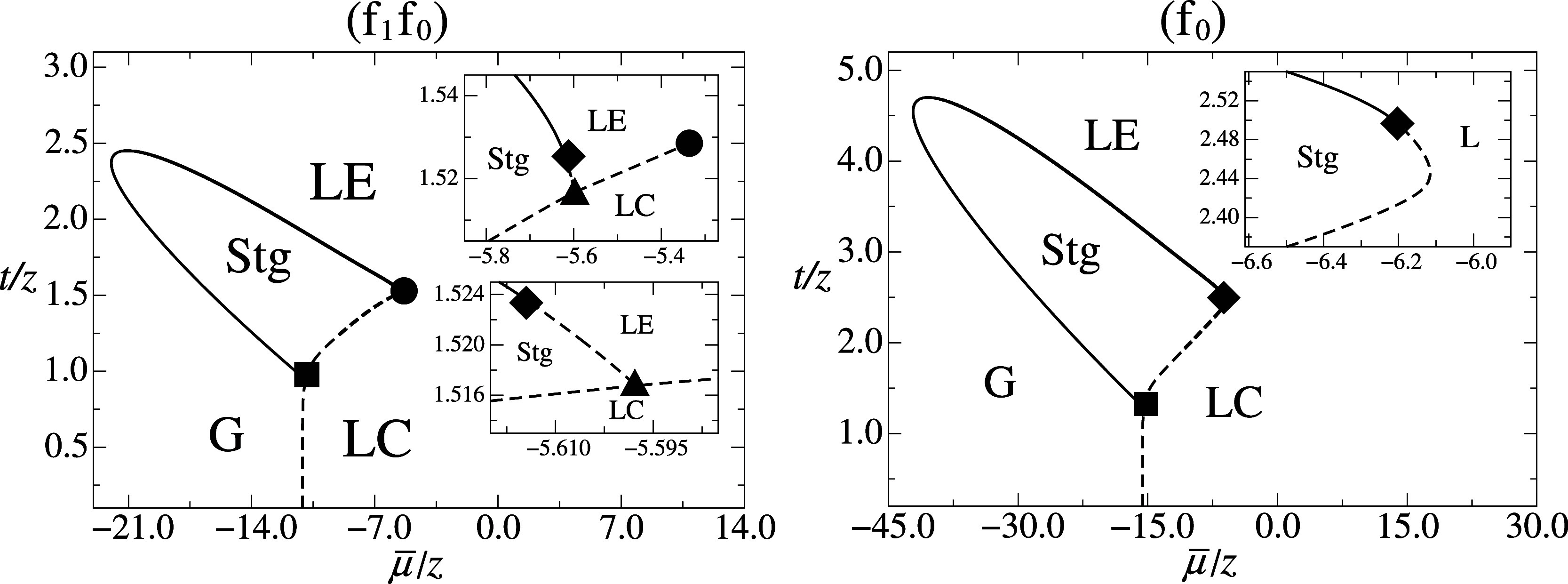
Dimensionless temperature *t*/*z* versus μ̅/*z* typical phase diagrams obtained for the DLG model under MFA for *h̅* = 0 and ω = 4 × 10^4^ for the intermediate subregions of region (f) presented in Figure 1. The pair values (k̅,l̅) corresponding to each region are given in Table 1. In addition to the standard (G, LE, LC) phases already present in the previous phase diagrams, there is also a Stg phase, whose phase transitions are associated with critical end points (■) and tricritical points (◆). In the case (f_0_) there is no transition line LE-LC, since no longer there is any distinction between the previous LE and LC phases, but we chose to keep the distinct nomenclature for better understanding in the major picture. The same representation introduced in Figure 1 for the thermodynamic phases, transition lines and symbols for the special points have been used here. The insets represent magnifications of the regions where the associated LE-LC first-order transition line ending at a critical point shrinks (f_1_f_0_) and where it ceases to exist (f_0_).

**Table 1:** Pair Values (k̅,l̅) Corresponding to the Typical Theoretical Phase Diagrams Displayed in Figures 2 and 3 ^a^

Region	*k̅*	l̅
(*t*/*z*)×(μ̅/*z*) Phase Diagrams		
b_1_	20.0	12.0
b_1_d_1_	14.25	11.05
b_2_d_2_	14.0	10.94
d_2_	10.9	11.12
a_1_c_1_	13.5	10.58
c_1_	13.4	10.58
f_1_f_0_	5.2	16.0
f_0_	5.2	25.0

DMPC Π×*T* Fittings		
MFA: d_1_	8.4514	10.8771
BPA: d_1_	6.51200	9.30161

a In addition, the parameters associated with the DMPC Π×*T* fittings for MFA and BPA, displayed in Figure 4, are listed. The fitting procedure is discussed in detail in the subsection “Theory Versus Experiments”.

For the old region (f) we have the new topologies (f_1_f_0_) and (f_0_) which, like the case (f_1_), present the Stg phase in addition to the standard uniform phases (Figure 3). In these new cases, there is additionally the occurrence of a tricritical point, where a first-order line turns into a second-order line. The analysis presented in Appendix III allowed us to locate this tricritical point. In the (f_1_f_0_) case (Figure 3 on the left), besides the LE-LC and Stg-LC first-order phase transitions, we have the Stg-LE first-order transition and the triple point Stg-LE-LC. By increasing the parameter l̅ , the first-order LE-LC phase transition shrinks, ceasing to be physically stable, and we enter the (f_0_) region, where the Stg-LC and Stg-LE transition lines become a single one (Stg-L), since there is no longer a distinction between the LC and LE phases (Figure 3 on the right). The boundary between the regions (f_1_) and (f_1_f_0_) is determined by the collapse condition of the tricritical point Stg-LE with the triple point Stg-LE-LC, i.e., when it also satisfies the first-order condition referring to the LC phase. The boundary between the regions (f_1_f_0_) and (f_0_) is determined by the condition of the collapse of the critical point LE-LC with the triple point Stg-LE-LC, i.e., with the first-order condition referring to the Stg phase. The boundary between the regions (d_1_) and (f_1_), as well as between the regions (c_0_) and (e_0_), is obtained by the collapse of the Stg phase and its two critical end points on the first-order Stg-LC transition line, associated to the additional condition presented in Appendix IV.

To better visualize the transformations that occur in the phase diagrams when crossing the boundaries of the various regions (Figure 1), we summarize them in Table 2. In order to locate the limits of the regions in the (*k̅*, l̅ ) diagram (Figure 1) and to understand certain collapses summarized in Table 2, it was necessary to obtain the multicritical conditions of the DLG model in MFA, extending the analysis presented in Appendix A of ref 34. This demands some cumbersome algebra and technical details, so the conditions associated to multicritical points and collapse of critical end points are presented in Appendices I to IV.

**Table 2:** Collapses of Critical Points (CP), Critical End Points (CEP) and Tricritical Points (TCP) into Triple Points (TP) or into Transition Lines,^a^ Associated with Topology Transformations in the Diagram k̅×l̅ (Figure 1)

Boundary	Associated Collapse
b_2_ → b_1_	CP LE-LC → TP G-LE-LC
b_1_d_1_ → b_1_	CP LE1-LE2 → TP G-LE1-LE2
b_1_d_1_ → d_1_	CP G-LE → TP G-LE1-LE2
d_2_ → d_1_ (on the left)	CP LE1-LE2 → TP LE1-LE2-LC
d_2_ → d_1_ (on the right)	CP LE-LC → TP LE1-LE2-LC
b_2_d_2_ → b_2_	CP LE1-LE2 → TP G-LE1-LE2
b_2_d_2_ → d_2_	CP G-LE → TP G-LE1-LE2
b_2_d_2_ → b_1_d_1_	CP LE-LC → TP G-LE-LC
a_1_c_1_ → a_1_	CP LE1-LE2 → TP LE1-LE2-LC
a_1_c_1_ → c_1_	CP G-LE → TP G-LE-LC
a_1_ → c_0_	CP G-LE → TP G-LE-LC
c_1_ → c_0_	CP LE1-LE2 → TP LE1-LE2-LC
e_0_ → c_0_	L2 Stg-G-LE CEP pair → L1 LE-LC
f_1_ → d_1_	L2 Stg-G-LE CEP pair → L1 LE-LC
f_1_f_0_ → f_1_	TCP Stg-LE → TP Stg-LE-LC
f_1_f_0_ → f_0_	CP LE-LC → TP Stg-LE-LC

a When necessary, we refer to first-order (L1) or second-order (L2) transition lines.

### Theory Versus Experiments

As performed in ref 35 for BPA, we can also compare our MFA theoretical predictions with experimental measurements of the commonly studied double-saturated zwitterionic phospholipid DMPC. In particular, we choose the same data sets, referring to the LE-LC phase-transition isotherms^48^—presented in Table 3—used in the previous comparison of this data with the results in BPA.^35^ So, the results of the experimental fitting performed in MFA can be also compared with the results in BPA.

**Table 3:** Experimental Data Extracted from Lateral Pressure Isotherms of Ref 48, Concerning the LC-LE First-Order Transition for DMPC Monolayers^a^

Π (mN/m)	*T* (°C)
21.7682	12
22.9849	13
25.4179	14
27.4935	15
31.2864	16
32.7892	17
34.5783	18
36.0100	19
Critical Point	
43.3160	20

a The last row of the table refers to the critical point (Π_c_, *T*_c_). Compiled from Table 1 of ref 35. Copyright 2019 American Chemical Society.

To adjust model parameters in order to reproduce the experimental values, we must be careful about how to relate the data taken from the experimental graphs with the corresponding theoretical variables. In our theoretical model, the total area is fixed and given by *A* = *Na*_0_, where *N* represents the total number of system sites, both empty—that is, occupied by water molecules—and those occupied by a lipid molecule. Therefore, the grand-canonical potential Ψ, related to the lateral pressure Π and total area *A* by the Euler relation, can be written as^35^

35 $$\Psi =\frac{N\psi }{\beta }=\frac{A\psi }{\beta a_{0}}=-\Pi A,\to ,\Pi =-\frac{\psi }{\beta a_{0}}$$

In the same way as proposed in ref 35, in order to obtain a better agreement in the comparison of experimental data with theoretical results, we introduced a correction factor that relates the MFA critical temperature to the exact critical temperature of the spin-1/2 ferromagnetic Ising model on a two-dimensional triangular lattice (*z* = 6), which is incorporated into the expression of the lateral pressure, Π→φΠ, with

36 $$\varphi =\frac{t_{c}^{\mathrm{MFA}}}{t_{c}^{\mathrm{exact}}\left(\Delta \right)}=\frac{z}{4/\ln3}\approx 1.64792...$$

The area *a*_0_, associated with the lattice parameter, was used coinciding with the area occupied by the phospholipid in the ordered state, *a*_0_ = *a*_o_ = 46.9 Å^2^.^35^ The DMPC phospholipids have two saturated 14-carbon tails, and the degeneracy parameter is estimated to be close to ω ≈ 4 × 10^4^.^51^ In addition, for simplicity the external field parameter was set to *h̅* = 0. The steps followed to adjust the model parameters were the same as those performed in ref 35. In Figure 4, we display the coexistence lateral pressure versus transition temperature Π×*T* phase diagram obtained under MFA and BPA and the experimental data (Table 3). As in BPA fitting, we found only a LE-LC first-order transition line ending at a critical point, in agreement with the experimental observations. By comparing the two theoretical curves, we observed that BPA is in slightly better agreement with the experimental data than MFA, as expected. It is noteworthy to mention that the leftward drift observed in the theoretical Π×*T* coexistence curves as improved approximations are employed and their presumably better agreement with experimental data represents a peculiarity of the DLG model. For the two-state Doniach model,^29^ e.g., all approximations collapse on the straight-line exact solution. Notice, furthermore, that the experimental DMPC Π×*T* coexistence data presented in Figure 4 display a nonmonotonic behavior, with a slightly inconsistent S shape.

**Figure 4:**
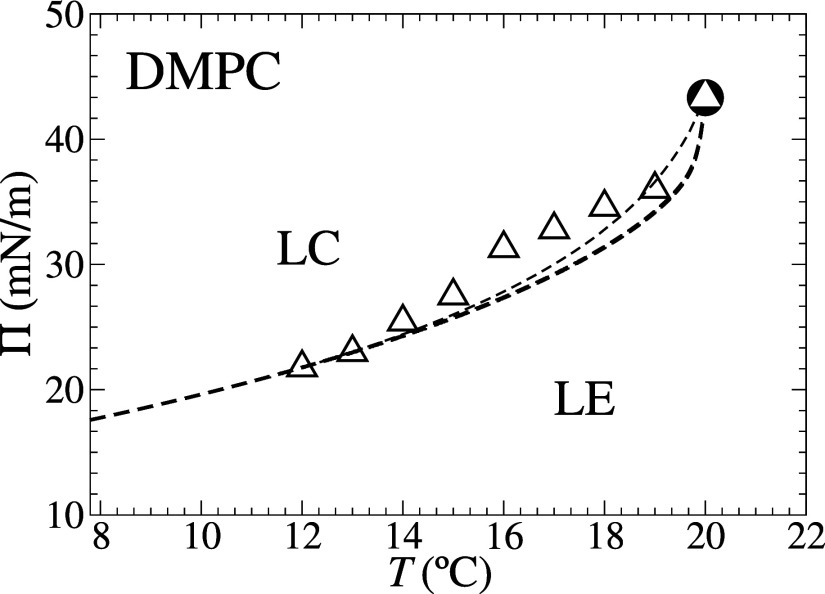
Temperature × coexistence lateral pressure phase diagram obtained for the DLG model under MFA (thick dashed line) and BPA (thin dashed line)^35^ using numerical parameters obtained by fitting experimental isothermal compression data corresponding to DMPC, represented by (△) and listed in Table 3.^48^ The dashed lines represent the first-order phase transition between the LE and the LC phases that ends at a critical point (●), obtained by the DLG model in MFA (*z* = 6, ω = 4 × 10^4^, *h̅* = 0, l̅ = 10.8771, *k̅* = 8.4514) and in BPA (*z* = 6, ω = 4 × 10^4^, *h̅* = 0, l̅ = 9.30161, *k̅* = 6.51200).

It should be remarked that the DLG model simplifies the occupied areas in the lattice by considering a fixed area per site *a*_0_ that is equivalent to the minimum area of the lipid-head ordered state *a*_0_ ≡ *a*_o_, which are attributed both to the water molecules and to the lipid heads, regardless of their state, ordered or disordered. Experimentally the area of the disordered state *a*_d_ varies greatly with temperature, while the vacant sites, mimicked in the DLG model by the same area *a*_0_, can actually correspond to a cluster of water molecules. In the DLG model the area per lipid head is defined by *a* ≡ *A*/⟨*N*_lip_⟩ = *a*_0_/*q* ≥ *a*_0_, depending thus on the order parameter *q* that measures the lipid density of the system. Therefore, the observed variation of *a*_d_ could be reproducible, in principle, by suitable changes in *q*. However, the DMPC LE areas estimated by using the obtained (k̅,l̅) fitting parameters are significantly larger at lower temperatures and smaller near the critical point than their experimental counterparts. When the theoretical pressure isothermal curves are plotted—not shown here, but see similar behavior found for BPA in Figure 7 of ref 35—one observes a disagreement between them and the experimental data. From a purely theoretical analysis, we should expect an overestimation of the LE area per lipid head *a*_d_, since the water-filled sites should occupy an actual area smaller than the fictitious area *a*_0_ attributed to them in the model. On the other hand, the underestimation of *a*_d_ near the critical conditions could be attributed to the MFA itself, although BPA^35^ also yields similar disagreeing results. To possibly improve the agreement between the results of the theoretical model and the experimental measurements, it might be necessary to consider a modified DLG model that takes into account the differences of the occupied areas by lipid heads and by water molecules. However, at first glance, the introduction of two distinct areas in the model gives rise to challenging technical problems related to the most suitable ensemble to perform the calculations. Attempts to implement this discrimination between areas corresponding to lipid heads and to water molecules may be subject of future work.

## Conclusions

We revisited the DLG model in MFA by considering it on a bipartite lattice. By splitting the system into two interpenetrating sublattices, it was possible to confirm the occurrence of the Stg phase at the MFA level for a certain range of parameters. In the original DLG paper,^33^ this range of parameters was overlooked and the Stg phase was first found only under BPA approximation.^34,35^ By revisiting the DLG model at MFA we prove that the occurrence of the Stg phase is not intrinsic to the chosen approximation. In addition, with further investigations, we obtained new topologies of (μ̅/*z*, *t*/*z*) phase diagrams, complementing the (l̅,k̅) diagram presented in ref 34. In particular, in some cases we also observed the occurrence of a discontinuous phase transition between two distinct disordered-chain (LE1-LE2) lipid phases.

For the range of interaction parameters in which the Stg phase occurs, the effective spin-1/2 Ising model that describes the G-LE transition becomes *antiferromagnetic*. As already mentioned in the concluding remarks of the BPA works,^34,35^ we reinforce that the ordered state of the spin-1/2 Ising antiferromagnet on a triangular lattice (*z* = 6) in the presence of an external magnetic field is nontrivial due to geometric frustration and cannot be simply described by a bipartite-lattice Stg state,^52–58^ as performed in this work. To properly analyze the DLG model in this case, we should consider a lattice that displays the appropriate sublattice geometry compatible with the (nontrivial) ordered state of the model at low temperatures, such as the Husimi cactus.^59–62^ It is expected that with a proper treatment on a tripartite lattice, the Stg single-lobe phase region in the (μ̅/*z*, *t*/*z*) phase diagrams, predicted for bipartite lattices, should be replaced by a double-lobe structure. Preliminary calculations of the DLG model on a tripartite Husimi cactus^63^ confirm this forecast, but the Stg phases turn possible only at negative values of *k̅* parameter and the transition becomes discontinuous. Furthermore, no indication of the coexistence of two distinct disordered-chain lipid phases was found on the Husimi-cactus calculations, which may suggest that the observed MFA intermediate phase-diagram topologies are spurious.

The concluding remarks of the BPA work^34^ suggested that the Stg phase might be related to the ripple phase, which appears in both zwitterionic and ionic bilayer systems.^10–15^ The connection between the two phases is not trivial and may not be feasible. The Stg phase in the DLG model arises from a certain combination of interaction parameters, namely for ϵ_wd_ > $\frac{1}{2}$ ­(ϵ_ww_ + ϵ_wo_), when the limiting spin-1/2 Ising model that describes the G-LE transition becomes antiferromagnetic. The possible comparison between the Stg phase and the ripple phase in bilayers involves tracking down a possible relationship with experimental data on bilayers. We could analyze the LC-Stg-LE sequence that occurs, for example, on the second inset to the left panel of Figure 3, by varying the temperature on isobaric (constant lateral pressure) lines (not shown here), based on the assumption that the lateral pressure remains constant in lipid-bilayer vesicles.^64^ With this analysis it is possible to verify that the main-transition in bilayers would correspond to the LE-LC transition and the pretransition would occur only at *higher temperatures*, which is inconsistent with the experimental heat-capacity data, that generally show a pretransition signature occurring at temperatures lower than the main-transition.^14,20,22,23^ For a proper description of the ripple phase in terms of an interaction model, specific properties of the intermediate phase may have to be considered and included in the model. To improve it, it would perhaps be necessary to consider different interaction terms, like competing next-nearest-neighbor interactions,^14,18,19^ and a complementary approach to describe curvature properties of the ripple phase by using continuum theories.^16,17,21,24^ Despite being a relevant point and one of our interest, it is beyond the scope of this paper to attempt this connection.

To complete this work we briefly present a comparison between the numerical results acquired through the theoretical model with experimental results present in the literature^48^ associated with the LC-LE transition in Langmuir monolayers of the zwitterionic phospholipid DMPC. This theory-versus-experiment comparison had been previously performed for the DLG model at the pair-approximation level through the Bethe−Gujrati method,^47^ implemented via calculations on a Cayley tree.^35^ Thus, by using the same experimental data as in these previous works, it was possible to compare the results obtained at the MFA level both with the experimental results directly and with the numerical results of the DLG model at the BPA level.

## Appendix I: Limits of Numerical Stability

In this Appendix, we obtain the additional conditions associated with the limits of numerical stability of the thermodynamic phases. These limits correspond to spinodal lines for uniform phases, and to critical boundaries for staggered phases on bipartite lattices.

The two equations of state associated with the order parameters in a uniform system, eqs 17 and 18, can be interpreted as a nonlinear mapping *m* = *m̅*(*m*, *q*; *h*, μ), *q* = *q̅*(*m*, *q*; *h*, μ), where *m̅* and *q̅* are the functions on the right-hand side of eqs 17 and 18, which were previously denoted simply as *m*(*h*, μ) and *q*(*h*, μ), respectively. The condition associated with the limits of numerical stability of the distinct numerical solutions of the equations of state can be obtained either by using the thermodynamic procedure presented in refs 37 and 65—to be also presented in the subsequent Appendices—or by looking at the eigenvalues of the Jacobian matrix associated with the nonlinear mapping (*m*, *q*) → (*m̅*, *q̅*),

37
$$J\left(m,q\right)\equiv \frac{\partial \left(\mathrm{m̅},\mathrm{q̅}\right)}{\partial \left(\eta ,\theta \right)}{\left(\frac{\partial \left(\eta ,\theta \right)}{\partial \left(m,q\right)}\right)}_{h,\mu }=\left(\begin{array}{cc} q-m^{2} & m\left(1-q\right)\\ m\left(1-q\right) & q\left(1-q\right) \end{array}\right)\left(\begin{array}{cc} j & \frac{1}{2}l\\ \frac{1}{2}l & k \end{array}\right)=\left(1-q\right)\left(\begin{array}{cc} \frac{j\left(q-m^{2}\right)}{1-q}+\frac{1}{2}lm & km+\frac{l\left(q-m^{2}\right)}{2\left(1-q\right)}\\ jm+\frac{1}{2}lq & kq+\frac{1}{2}lm \end{array}\right)$$

The spinodal-line condition for uniform phases are associated with the eigenvalue λ = 1 of the Jacobian matrix J(m,q) , which can be solved for the *k* parameter to yield

38
$$k_{CP}^{\left(1\right)}=\frac{\left(1-q\right)l\left[l\left(q^{2}-m^{2}\right)+4m\right]-4-4j\left(m^{2}-q\right)}{4\left(1-q\right)\left[j\left(q^{2}-m^{2}\right)-q\right]}$$

A critical point corresponding to the *terminus* of a first-order transition line between two uniform phases is located at the meeting of the spinodal lines of the two phases undergoing the first-order transition. In the next Appendix we obtain the additional condition *k*_CP_^(2)^ that defines the critical value of *k*, eqs 44 and 60.

For a bipartite lattice, notice that the equations of state can be cast in the form of a coupled mapping, *m_a_* = *m̅*(*m_b_*, *q_b_*; *h*, μ), *q_a_* = *q̅*(*m_b_*, *q_b_*; *h*, μ), and *m_b_* = *m̅*(*m_a_*, *q_a_*; *h*, μ), *q_b_* = *q̅*(*m_a_*, *q_a_*; *h*, μ), by using the same nonlinear mapping (*m̅*, *q̅*) defined by eqs 17 and 18. In this case, one needs to investigate the eigenvalues of the product of the Jacobian matrices $J\left(m_{a}\;,q_{a}\right)J\left(m_{b}\;,q_{b}\right)$ , the spinodal condition being associated again with the eigenvalue λ = 1 of the product matrix. Therefore, to locate the spinodal line of a Stg phase that undergoes a first-order transition would require this calculation. On the other hand, the critical line Stg-LE (or Stg-G) corresponds to the case when the solutions corresponding to the two sublattices collapse continuously, *m*_*a*_→*m*_*b*_, *q*_*a*_→*q*_*b*_. In this case, the limit of stability coincides with the critical line, and it is associated with the eigenvalue λ = −1 of one of the two identical Jacobian matrices, J(m,q) = $J\left(m_{a}\;,q_{a}\right)$ = $J\left(m_{b}\;,q_{b}\right)$ , which can again be solved for the *k* parameter to yield

39
$$k_{CL}=\frac{\left(1-q\right)l\left[l\left(q^{2}-m^{2}\right)-4m\right]-4+4j\left(m^{2}-q\right)}{4\left(1-q\right)\left[j\left(q^{2}-m^{2}\right)+q\right]}$$

In Appendix III we show that this critical condition may be also obtained by a Landau-like expansion of the Helmholtz free-energy density *f*(***m***, ***q***) on bipartite lattices, eq 75.

## Appendix II: Multicritical Conditions Involving Only Uniform Phases

In this Appendix, we obtain the conditions for the multicritical points involving only uniform phases.

It is convenient to define the dimensionless Helmholtz free-energy density per site *f*(*m*, *q*) ≡ β*F/N*, obtained by an inverse Legendre transform of ψ­(*h*, μ), in order to eliminate the conjugated fields (*h*, μ)

40
$$f\left(m,q\right)=\max_{h,\mu }\left[\psi \left(h,\mu \right)+mh+q\mu \right]=\psi \left[h\left(m,q\right),\mu \left(m,q\right)\right]+mh\left(m,q\right)+q\mu \left(m,q\right)=-\frac{1}{2}\left(jm^{2}+kq^{2}+lmq\right)-\frac{1}{2}\left(q-m\right)\;\ln\omega +\left(1-q\right)\;\ln\left(1-q\right)+\frac{1}{2}\left(q+m\right)\;\ln\left[\frac{1}{2}\left(q+m\right)\right]+\frac{1}{2}\left(q-m\right)\;\ln\left[\frac{1}{2}\left(q-m\right)\right]$$

One method to locate the multicritical points corresponding to the *terminus* of a first-order transition line between two uniform phases is the thermodynamic procedure presented in refs 37 and 65 . The critical conditions are related to the vanishing of the partial derivatives *h* ^(1)^ = *h* ^(2)^ = 0, where *h* ^(*n*)^ is the *n*-th partial derivative of the field *h* conjugated to *m*, for constant μ, defined by

41
$$h^{\left(n\right)}\equiv {\left(\frac{\partial^{n}h}{\partial m^{n}}\right)}_{\mu }={\left(\frac{\partial^{n}f_{m}}{\partial m^{n}}\right)}_{f_{q}}=\frac{1}{f_{2q}},\frac{\partial \left(h^{\left(n-1\right)},f_{q}\right)}{\partial \left(m,q\right)}$$

For the sake of a clean notation, we used a condensed notation for the partial derivatives of *f*(*m*, *q*)­

42
h=fm≡(∂f∂m)q,μ=fq≡(∂f∂q)m,f2m≡(∂fm∂m)q,f2q≡(∂fq∂q)m,fmq≡(∂fm∂q)m=fqm,...(42)

Therefore, by using computer software that can perform algebraic manipulations, we can obtain the necessary partial derivatives of *f*(*m*, *q*) up to third order, and the critical-point conditions involving uniform phases can be cast explicitly by the simultaneous eqs 43 and 44:

43
h(1)=f2mf2q−fmq2f2q=0⁣→kCP(1)=(1−q)l[l(q2−m2)+4m]−4−4j(m2−q)4(1−q)[j(q2−m2)−q](43)

44
h(2)=f3m−3fmqf2mqf2q+3(fmqf2q)2fm2q−(fmqf2q)3f3q=0→kCP(2)=κ1κ2(44)

45
$$\kappa_{1}=m^{4}\left[8jq-4j^{2}\left(4q^{2}-2q+1\right)-3l^{2}{\left(1-q\right)}^{2}\right]+4j^{2}m^{6}+4lm^{3}\left(1-q\right)\left\{3-q\left[3j\left(1-q\right)+2\right]\right\}+q^{2}\left\{4+q[4j^{2}\left(1-2q\right)q+8j\left(2q-1\right)+3l^{2}{\left(1-q\right)}^{2}q-8]\right\}-4lm\left(1-q\right)q^{2}\left[1-3j\left(1-q\right)q\right]-4m^{2}\left\{3-q\left[4+j\left(3jq^{3}-8q+4\right)\right]\right\}$$

46
$$\kappa_{2}=4m{\left(1-q\right)}^{2}q\left(lm^{2}-lq^{2}-2m\right)$$

Notice that eq 43 represents indeed the spinodal-line condition, and coincides with eq 38, obtained by the eigenvalue λ = 1 of the Jacobian matrix J(m,q) , eq 37.

Proceeding with the calculations in order to obtain the additional tricritical conditions, we obtain eqs A3 and A4 of ref 65,

47
$$h^{\left(3\right)}=0,\to ,f_{2m}^{3}f_{4q}-4f_{2m}^{2}f_{mq}f_{m3q}+6f_{mq}^{2}f_{2m}f_{2m2q}-4f_{mq}^{3}f_{3mq}+f_{mq}^{2}f_{2q}f_{4m}-3f_{2m}^{2}f_{m2q}^{2}-3f_{mq}^{2}f_{2mq}^{2}+3(f_{mq}^{2}f_{m2q}-f_{2m}f_{mq}f_{3q})f_{3m}+3f_{2m}^{2}f_{2mq}f_{3q}+3f_{2m}f_{mq}f_{2mq}f_{m2q}=0$$

48
$$h^{\left(4\right)}=0,\to ,f_{2m}^{4}f_{5q}-5f_{2m}^{3}f_{mq}f_{m4q}+10f_{mq}^{2}f_{2m}^{2}f_{2m3q}-10f_{mq}^{3}f_{2m}f_{3m2q}+5f_{mq}^{4}f_{4mq}-f_{mq}^{3}f_{2q}f_{5m}+\left(4f_{2m}f_{3q}-2f_{mq}f_{m2q}\right)f_{mq}^{2}f_{4m}+\left[12f_{mq}f_{2m}f_{m2q}^{2}-3f_{mq}^{2}f_{2mq}f_{m2q}-3f_{2m}^{2}f_{3q}f_{m2q}+14f_{mq}^{2}f_{2m}f_{m3q}-12f_{2m}f_{mq}f_{2mq}f_{3q}-6f_{2m}^{2}f_{mq}f_{4q}+2f_{mq}^{2}f_{2q}f_{3mq}+3f_{mq}^{2}f_{3m}f_{3q}\right]f_{3m}+8f_{2m}^{3}f_{2mq}f_{4q}-12f_{2m}^{2}f_{mq}f_{2mq}f_{m3q}-10f_{2m}^{3}f_{m2q}f_{m3q}+48f_{2m}^{2}f_{mq}f_{m2q}f_{2m2q}-6f_{2m}^{3}f_{3q}f_{2m2q}-30f_{2m}f_{mq}^{2}f_{2mq}f_{2m2q}-6f_{mq}^{2}f_{m2q}f_{3mq}f_{2m}+4f_{mq}^{3}f_{2mq}f_{3mq}-8f_{2m}^{2}f_{mq}f_{3q}f_{3mq}+12f_{2m}^{2}f_{3q}f_{2mq}^{2}-9f_{2m}^{2}f_{2mq}f_{m2q}^{2}=0$$

Notice that, apparently, there is a misprint in ref 65, since the contribution 3*f*_*mq*_^2^ *f*_*m*2*q*_ *f*_3*m*_ in eq 47 reads (incorrectly) 3*f*_*mq*_^3^ *f*_*m*2*q*_ *f*_3*m*_ in eq A3 of ref 65. However, for the range of Hamiltonian parameters investigated here, these two conditions were never fulfilled at the critical points involving uniform phases.

As it is not clear how the above procedure, presented in refs 37 and 65 , can be later generalized in order to obtain the tricritical conditions involving transitions with Stg phases, we present an alternate, but equivalent, method to obtain the multicritical conditions based on a Landau-like expansion of the equations of state and of the Helmholtz free-energy density. We expand the equations of state (*h*, μ) ≡ (*f*_*m*_, *f*_*q*_) in the Helmholtz representation, eqs 19 and 20, about the paramagnetic solution (*m* + δ, *q* + ϵ), with the *Ansatz*

49
$$\epsilon =a\delta +b\delta^{2}+c\delta^{3}+d\delta^{4}+O\left(\delta^{5}\right)$$

for the relationship between the expansion parameters (δ, ϵ). The set of expansion coefficients (*a*, *b*, *c*, *d*) are obtained by self-consistency of the equations of state,

50
$$a=-\frac{f_{mq}}{f_{2q}}$$

51
$$b=-\frac{1}{2f_{2q}}\left(f_{2mq}+2af_{m2q}+a^{2}f_{3q}\right)$$

52
$$c=-\frac{b}{f_{2q}}\left(f_{m2q}+af_{3q}\right)-\frac{1}{6f_{2q}}\left(f_{3mq}+3af_{2m2q}+3a^{2}f_{m3q}+a^{3}f_{4q}\right)$$

53
$$d=-\frac{c}{f_{2q}}f_{m2q}-\frac{1}{2f_{2q}}[b\left(f_{2m2q}+2af_{m3q}+a^{2}f_{4q}\right)+\left(b^{2}+2ac\right)f_{3q}]-\frac{1}{24f_{2q}}\left(f_{4mq}+4af_{3m2q}+6a^{2}f_{2m3q}+4a^{3}f_{m4q}+a^{4}f_{5q}\right)$$

and the multicritical conditions are associated with the vanishing of the {*B*_*n*_} coefficients of the Taylor expansion of the Helmholtz free-energy density *f*(*m*, *q*) about the paramagnetic solution,

54
$$f\left(m+\delta ,q+\epsilon \right)=f\left(m,q\right)+\left(\delta ,\epsilon \right)\cdot \left(h,\mu \right)+\sum_{n=2}^{5}B_{n}\delta^{n}+O\left(\delta^{6}\right)$$

55
$$B_{2}\equiv \frac{1}{2}\left(f_{2m}+2af_{mq}+a^{2}f_{2q}\right)=\frac{1}{2}\left(f_{2m}-\frac{f_{mq}^{2}}{f_{2q}}\right)$$

56
$$B_{3}\equiv b\left(f_{mq}+af_{2q}\right)+\frac{1}{6}\left(f_{3m}+3af_{2mq}+3a^{2}f_{m2q}+a^{3}f_{3q}\right)$$

57
$$B_{4}\equiv cf_{mq}+\frac{1}{2}\left[b\left(f_{2mq}+2af_{m2q}+a^{2}f_{3q}\right)+\left(b^{2}+2ac\right)f_{2q}\right]+\frac{1}{24}\left(f_{4m}+4af_{3mq}+6a^{2}f_{2m2q}+4a^{3}f_{m3q}+a^{4}f_{4q}\right)$$

58
$$B_{5}\equiv df_{mq}+\left(ad+bc\right)f_{2q}+\frac{1}{2}\left[a\left(b^{2}+ac\right)f_{3q}+\left(b^{2}+2ac\right)f_{m2q}+cf_{2mq}\right]+\frac{1}{6}b\left(f_{3mq}+3af_{2m2q}+3a^{2}f_{m3q}+a^{3}f_{4q}\right)+\frac{1}{120}\left(f_{5m}+5af_{4mq}+10a^{2}f_{3m2q}+10a^{3}f_{2m3q}+5a^{4}f_{m4q}+a^{5}f_{5q}\right)$$

Now the critical-point conditions are given by *B*_2_ = *B*_3_ = 0, while the tricritical-point conditions read *B*_2_ = *B*_3_ = *B*_4_ = *B*_5_ = 0. The conditions expressed in terms of the {*B*_*n*_} coefficients allow us to explicitly solve the *k* = *k* ^(*n*)^ parameters that vanish the respective coefficients *B*_*n*+1_,

59
$$k_{CP}^{\left(1\right)}=\frac{1}{1-q}+\frac{1}{2}\left(\frac{1}{q+m}+\frac{1}{q-m}\right)-\frac{f_{mq}^{2}}{f_{2m}}$$

60
$$k_{CP}^{\left(2\right)}=\frac{1}{1-q}+\frac{1}{2}\left(\frac{1}{q+m}+\frac{1}{q-m}\right)+\frac{1}{a}f_{mq}+\frac{1}{6ab}\left(f_{3m}+3af_{2mq}+3a^{2}f_{m2q}+a^{3}f_{3q}\right)$$

61
$$k_{TCP}^{\left(3\right)}=\frac{1}{1-q}+\frac{1}{2}\left(\frac{1}{q+m}+\frac{1}{q-m}\right)+\frac{1}{b^{2}+2ac}\left[2cf_{mq}+b\left(f_{2mq}+2af_{m2q}+a^{2}f_{3q}\right)+\frac{1}{12}\left(f_{4m}+4af_{3mq}+6a^{2}f_{2m2q}+4a^{3}f_{m3q}+a^{4}f_{4q}\right)\right]$$

62
$$k_{TCP}^{\left(4\right)}=\frac{1}{1-q}+\frac{1}{2}\left(\frac{1}{q+m}+\frac{1}{q-m}\right)+\frac{1}{ad+bc}\{df_{mq}+\frac{1}{2}[a\left(b^{2}+ac\right)f_{3q}+\left(b^{2}+2ac\right)f_{m2q}+cf_{2mq}]+\frac{1}{6}b\left(f_{3mq}+3af_{2m2q}+3a^{2}f_{m3q}+a^{3}f_{4q}\right)+\frac{1}{120}\left(f_{5m}+5af_{4mq}+10a^{2}f_{3m2q}+10a^{3}f_{2m3q}+5a^{4}f_{m4q}+a^{5}f_{5q}\right)\}$$

obtained by solving the conditions *B*_*n*_ = 0 for *f*_2*q*_, and replacing the occurrences of *f*_2*q*_ = *f*_*mq*_^2^/*f*_2*m*_ into the expressions of the (*a*, *b*, *c*, *d*) coefficients. These closed analytical expressions for the multicritical values of the *k* parameter are fully equivalent to the results previously reported in ref 65, and also eqs 43 and 44 for the critical conditions.

## Appendix III: Multicritical Conditions for Staggered Phases on Bipartite Lattices

In this Appendix, we obtain the multicritical conditions for transitions involving Stg phases on bipartite lattices, that can be obtained, in a more suitable form, by using a Landau-like expansion of the equations of state in the Helmholtz representation.

Again, in order to obtain the dimensionless Helmholtz free-energy density per site *f*(***m***, ***q***) ≡ β*F*/*N* for a bipartite lattice, we perform the inverse Legendre transform of ψ­(***h***, **μ**), eq 22, in order to eliminate the conjugated fields (***h***, **μ**),

63
$$f\left(m,q\right)=\max_{h,\mu }\left[\psi \left(h,\mu \right)+m\cdot h+q\cdot \mu \right]=\psi \left[h\left(m,q\right),\mu \left(m,q\right)\right]+m\cdot h\left(m,q\right)+q\cdot \mu (m,q)=-\frac{1}{2}\left(jm_{a}m_{b}+kq_{a}q_{b}\right)-\frac{1}{4}l\left(m_{a}q_{b}+m_{b}q_{a}\right)-\frac{1}{4}\left(q_{a}-m_{a}\right)\;\ln\omega -\frac{1}{4}\left(q_{b}-m_{b}\right)\;\ln\omega +\frac{1}{2}\left(1-q_{a}\right)\;\ln\left(1-q_{a}\right)+\frac{1}{2}\left(1-q_{b}\right)\;\ln\left(1-q_{b}\right)+\frac{1}{4}\left(q_{a}+m_{a}\right)\;\ln\left[\frac{1}{2}\left(q_{a}+m_{a}\right)\right]+\frac{1}{4}\left(q_{b}+m_{b}\right)\;\ln\left[\frac{1}{2}\left(q_{b}+m_{b}\right)\right]+\frac{1}{4}\left(q_{a}-m_{a}\right)\;\ln\left[\frac{1}{2}\left(q_{a}-m_{a}\right)\right]+\frac{1}{4}\left(q_{b}-m_{b}\right)\;\ln\left[\frac{1}{2}\left(q_{b}-m_{b}\right)\right]$$

At this point, it is convenient to introduce the variables associated with the global ***x*** ≡ (*m*, *q*) and staggered ***x***_s_ ≡ (*m*_s_, *q*_s_) order parameters, as well as their conjugate thermodynamic fields ***y*** ≡ (*h*, μ) = **∇⃗** *f* and ***y***_s_ ≡ (*h*_s_, μ_s_) = **∇⃗**_s_ *f*,

64
m≡12(ma+mb)=−(∂ψ∂h)hs,μ,q≡12(qa+qb)=−(∂ψ∂μ)h,μ s(64)ms≡12(ma−mb)=−(∂ψ∂hs)h,μ,qs≡12(qa−qb)=−(∂ψ∂μ s)h,μ(65)h≡12(ha+hb),⁣μ≡12(μ a+μb),hs≡12(ha−hb),⁣μ s≡12(μ a−μb)(66)

in terms of which the dimensionless Helmholtz free-energy density per site *f*(***x***, ***x***_s_) reads

67
$$f\left(x,x_{s}\right)=-\frac{1}{2}j\left(m^{2}-m_{s}^{2}\right)-\frac{1}{2}k\left(q^{2}-q_{s}^{2}\right)-\frac{1}{2}l\left(mq-m_{s}q_{s}\right)-\frac{1}{2}\left(q-m\right)\;\ln\omega +\frac{1}{2}\left(1-q-q_{s}\right)\;\ln\left(1-q-q_{s}\right)+\frac{1}{2}\left(1-q+q_{s}\right)\;\ln\left(1-q+q_{s}\right)+\frac{1}{4}\left(q+m+q_{s}+m_{s}\right)\;\ln\left[\frac{1}{2}\left(q+m+q_{s}+m_{s}\right)\right]+\frac{1}{4}\left(q+m-q_{s}-m_{s}\right)\;\ln\left[\frac{1}{2}\left(q+m-q_{s}-m_{s}\right)\right]+\frac{1}{4}\left(q-m+q_{s}-m_{s}\right)\;\ln\left[\frac{1}{2}\left(q-m+q_{s}-m_{s}\right)\right]+\frac{1}{4}\left(q-m-q_{s}+m_{s}\right)\;\ln\left[\frac{1}{2}\left(q-m-q_{s}+m_{s}\right)\right]$$

As the equations of state for **δ*x*** ≡ ***x*** – (*m*, *q*) represent even functions of *m*_s_, while those for ***x***_s_ represent odd functions of *m*_s_, when considering the Taylor expansions of the equations of state (***x***, ***x***_s_) around the paramagnetic solution in the vicinity of the critical condition, (***x***, ***x***_s_) = (*m* + δ, *q* + ϵ, *m*_s_, *q*_s_), the dominant terms of the expansion must assume the form

68
δ=a′ms2+O(ms4),⁣ϵ=b′ms2+O(ms4),qs=c′ms+d′ms3+O(ms5)(68)

Replacing these *Ansätze* on the Taylor expansion of the equations of state in the Helmholtz representation for the conjugate thermodynamic fields (***y***, ***y***_s_) around of the paramagnetic solution (*m* + δ, *q* + ϵ, *m*_s_, *q*_s_), we obtain the set (*a*′, *b*′, *c*′, *d*′) of coefficients by self-consistency of the equations of state,

69
(a′b′)=−12(f2mf2q−fmq2)×⁣(f2q−fmq−fmqf2m)(fm2ms+2c′fmmsqs+c′2fm2qsfq2ms+2c′fqmsqs+c′2fq2qs)(69)c′=−fmsqsf2qs(70)d′=−1f2qs[a′(fmmsqs+c′fm2qs)+b′(fqmsqs+c′fq2qs)⁣+16(f3msqs+3c′f2ms2qs+3c′2fms3qs+c′3f4qs)](71)

One should remark that all partial derivatives above of *f*(***x***, ***x***_s_) are evaluated at ***x*** = (*m*, *q*) and ***x***_s_ = (0, 0).

In the same way as for the uniform system, the multicritical conditions are associated with the vanishing of the even-power {*B*_*n*_^′^} coefficients of the Taylor expansion of the Helmholtz free-energy density *f*(***x***, ***x***_s_) about the paramagnetic solution ***x***_s_ = **0**,

72
$$f\left(x,x_{s}\right)=f\left(x,0\right)+\delta x\cdot y+B_{2}^{\prime }m_{s}^{2}+B_{4}^{\prime }m_{s}^{4}+O\left(m_{s}^{6}\right)$$

73
$$B_{2}^{\prime }\equiv \frac{1}{2}\left(f_{2m_{s}}+2c^{\prime }f_{m_{s}q_{s}}+{c^{\prime }}^{2}f_{2q_{s}}\right)=\frac{1}{2}\left(f_{2m_{s}}-\frac{f_{m_{s}q_{s}}^{2}}{f_{2q_{s}}}\right)$$

74
$$B_{4}^{\prime }\equiv \frac{1}{2}a^{\prime }\left(f_{m2m_{s}}+2c^{\prime }f_{mm_{s}q_{s}}+{c^{\prime }}^{2}f_{m2q_{s}}\right)+\frac{1}{2}b^{\prime }\left(f_{q2m_{s}}+2c^{\prime }f_{qm_{s}q_{s}}+{c^{\prime }}^{2}f_{q2q_{s}}\right)+\frac{1}{2}\left({a^{\prime }}^{2}f_{2m}+2a^{\prime }b^{\prime }f_{mq}+{b^{\prime }}^{2}f_{2q}\right)+d^{\prime }\left(f_{m_{s}q_{s}}+c^{\prime }f_{2q_{s}}\right)+\frac{1}{24}\left(f_{4m_{s}}+4c^{\prime }f_{3m_{s}q_{s}}+6{c^{\prime }}^{2}f_{2m_{s}2q_{s}}+4{c^{\prime }}^{3}f_{m_{s}3q_{s}}+{c^{\prime }}^{4}f_{4q_{s}}\right)$$

because the odd-power coefficients vanish trivially, *B*_3_^′^ = *B*_5_^′^ = 0.

The Stg-LE (or Stg-G) critical line condition *B*_2_^′^ = 0 can be cast in the form

75
$$f_{2q_{s}}=k_{CL}+\frac{1}{1-q}+\frac{1}{2}\left(\frac{1}{q+m}+\frac{1}{q-m}\right)=\frac{f_{m_{s}q_{s}}^{2}}{f_{2m_{s}}}=\frac{\frac{1}{4}{\left(l+\frac{1}{q+m}-\frac{1}{q-m}\right)}^{2}}{j+\frac{1}{2}\left(\frac{1}{q+m}+\frac{1}{q-m}\right)}$$

and it is satisfied by *k*_CL_ previously obtained in eq 39, through the eigenvalue λ = – 1 of the Jacobian matrix J(m,q) .

Making the substitution *f*_2*q*_s__ = *f*_*m*_s_ *q*_s__^2^/*f*_2*m*_s__, valid at the critical-line condition, and eliminating the partial derivatives that are identical, the tricriticality condition *B*_4_^′^ = 0 can be written in the form *A* + *Bf*_2*q*_ = 0, with coefficients

76
$$A\equiv 3f_{2m}{\left(f_{m_{s}q_{s}}^{2}f_{mm_{s}q_{s}}-2f_{2m_{s}}f_{m_{s}q_{s}}f_{qm_{s}q_{s}}+f_{2m_{s}}^{2}f_{q2q_{s}}\right)}^{2}+f_{mq}\{f_{m_{s}q_{s}}^{4}\left(f_{mq}f_{4m_{s}}-6f_{mm_{s}q_{s}}f_{qm_{s}q_{s}}\right)+f_{2m_{s}}^{4}\left(f_{mq}f_{4q_{s}}-6f_{qm_{s}q_{s}}f_{q2q_{s}}\right)+6f_{2m_{s}}^{2}f_{m_{s}q_{s}}^{2}\left[f_{mq}f_{4m_{s}}-f_{qm_{s}q_{s}}\left(5f_{mm_{s}q_{s}}+f_{q2q_{s}}\right)\right]+4\left(f_{2m_{s}}f_{m_{s}q_{s}}^{3}+f_{2m_{s}}^{3}f_{m_{s}q_{s}}\right)\left(3f_{qm_{s}q_{s}}^{2}-f_{mq}f_{m_{s}3q_{s}}\right)+12f_{2m_{s}}f_{m_{s}q_{s}}f_{mm_{s}q_{s}}\left(f_{m_{s}q_{s}}^{2}f_{mm_{s}q_{s}}+f_{2m_{s}}^{2}f_{q2q_{s}}\right)\}$$

77
$$B\equiv 3{\left[\left(f_{2m_{s}}^{2}+f_{m_{s}q_{s}}^{2}\right)f_{qm_{s}q_{s}}-2f_{2m_{s}}f_{m_{s}q_{s}}f_{mm_{s}q_{s}}\right]}^{2}-f_{2m}[\left(6f_{2m_{s}}^{2}+f_{m_{s}q_{s}}^{2}\right)f_{4m_{s}}f_{m_{s}q_{s}}^{2}-4\left(f_{2m_{s}}^{2}+f_{m_{s}q_{s}}^{2}\right)f_{2m_{s}}f_{m_{s}q_{s}}f_{m_{s}3q_{s}}+f_{2m_{s}}^{4}f_{4q_{s}}]$$

The explicit expression of the tricritical parameter *k*_TCP_(*j*, l ; *m*, *q*) can be obtained by solving the above condition for *f*_2*q*_, yielding

78
$$k_{TCP}=-f_{2q}+\frac{1}{1-q}+\frac{1}{2}\left(\frac{1}{q+m}+\frac{1}{q-m}\right)=\frac{A}{B}+\frac{1}{1-q}+\frac{1}{2}\left(\frac{1}{q+m}+\frac{1}{q-m}\right)$$

## Appendix IV: Condition for the Collapse of Critical End Points into the LE-LC First-Order Line

In this Appendix, we obtain the condition for the collapse of the critical end points into the first-order Stg-LC transition line. In order to determine the boundaries between the regions (e_0_)–(c_0_) and (f_1_)–(d_1_) i.e., the limit at which the phase Stg ceases to be present in the phase diagrams, we need to determine the condition at which the two critical end points of the edge of the Stg phase collapse into a single point on the LE-LC first-order line. If we plot the critical line *k* = *k*_CL_ coinciding with the critical end points as a function of μ̅, we notice that the target limit corresponds to the maximum of the curve *k*_CL_(μ̅, *j*), constrained to belong to the first-order line. Therefore, the constrained maximum of *k*_CL_, eq 39, can be more easily determined by maximizing the auxiliary function φ­(μ̅, *j*), obtained by adding a Lagrange-multiplier term to consider the constraint imposed by the first-order line,

79
$$\varphi \left(\mathrm{\mu ̅},j\right)\equiv k_{CL}+\Lambda \left(\psi -\psi^{\prime }\right)=k_{CL}\left(j,l;m,q\right)+\Lambda \left[\psi \left(j,k,l;m,q\right)-\psi^{\prime }\left(j,k,l;m^{\prime },q^{\prime }\right)\right]$$

where Λ is the Lagrange multiplier associated with the first-order transition between the uniform LE (*m*, *q*) and LC (*m*′, *q*′) phases, whose grand-canonical potentials are denoted by ψ and ψ′, respectively, and whose associated Helmholtz free energies are *f* = ψ + *mh* + *q*μ and *f*′ = ψ′ + *m*′*h* + *q*′μ. Eliminating the Lagrange multiplier at the extremization of the auxiliary function φ­(μ̅, *j*) leads us to the condition

80
$${\left(\frac{\partial \varphi }{\partial \mathrm{\mu ̅}}\right)}_{j}={\left(\frac{\partial \varphi }{\partial j}\right)}_{\mathrm{\mu ̅}}=0,\to ,{\left(\frac{\partial k_{CL}}{\partial \mathrm{\mu ̅}}\right)}_{j}{\left(\frac{\partial \left(\psi -\psi^{\prime }\right)}{\partial j}\right)}_{\mathrm{\mu ̅}}={\left(\frac{\partial k_{CL}}{\partial j}\right)}_{\mathrm{\mu ̅}}{\left(\frac{\partial \left(\psi -\psi^{\prime }\right)}{\partial \mathrm{\mu ̅}}\right)}_{j}$$

where ψ – ψ′ = *f* – *f*′ + (*m*′ – *m*)*h* + (*q*′ – *q*)­μ. As the above partial derivatives involve the uniform LE and LC phases, one may use the uniform-system Helmholtz free-energy density *f*, eq 40. For null external field (*h* = 0), we can write, from eqs 19 and 20,

81
$$h=0,\to ,j=\frac{1}{2m+lq\;/j}\left[\ln\left(\frac{q+m}{q-m}\right)+\ln\omega \right]$$

82
μ=(∂f∂q)m=−kq−12lm−12ln⁡ω+12ln(q+m)⁣+12ln(q−m)−ln[2(1−q)](82)

which allows us to obtain the Jacobian determinant

83
$$\frac{\partial \left(\mu ,j\right)}{\partial \left(m,q\right)}=\left(\begin{array}{cc} {\left(\frac{\partial \mu }{\partial m}\right)}_{q} & {\left(\frac{\partial \mu }{\partial q}\right)}_{m}\\ {\left(\frac{\partial j}{\partial m}\right)}_{q} & {\left(\frac{\partial j}{\partial q}\right)}_{m} \end{array}\right)=\left(\begin{array}{cc} -\frac{2m+l\left(q^{2}-m^{2}\right)}{2\left(q^{2}-m^{2}\right)} & \frac{q-m^{2}-k\left(1-q\right)\left(q^{2}-m^{2}\right)}{\left(1-q\right)\left(q^{2}-m^{2}\right)}\\ \frac{2j\left[q-j\left(q^{2}-m^{2}\right)\right]}{\left(2jm+lq\right)\left(q^{2}-m^{2}\right)} & -\frac{j\left[2m+l\left(q^{2}-m^{2}\right)\right]}{\left(2jm+lq\right)\left(q^{2}-m^{2}\right)} \end{array}\right)$$

suitable for the calculation of the collapse condition ([Disp-formula eq80]). Evaluating and simplifying the remaining partial derivatives, the final result for the collapse condition of the critical end points into the LE-LC coexistence line reads, for *h* = 0,

84
$$\frac{\partial \left(k_{CL},j\right)}{\partial \left(m,q\right)}\left\{\frac{1}{2}\left[j\left(m^{2}-m^{\prime 2}\right)+k\left(q^{2}-{q^{\prime }}^{2}\right)+l\left(mq-m^{\prime }q^{\prime }\right)\right]+\mu \left(q-q^{\prime }\right)\right\}=\left(q-q^{\prime }\right)\times \left\{\left[j\left(\frac{\partial k_{CL}}{\partial j}\right)+l\left(\frac{\partial k_{CL}}{\partial l}\right)\right]\frac{\partial \left(\mu ,j\right)}{\partial \left(m,q\right)}-j\frac{\partial \left(k_{CL},\mu \right)}{\partial \left(m,q\right)}\right\}$$

This expression can be rewritten as a linear equation *Ck* + *D* = 0, with coefficients (*C*, *D*) functions of the parameters (*j*, l ) and (*m*, *q*, *m*′, *q*′). The explicit forms of the coefficients are quite long and for this reason they are not shown, but they can be calculated by using an algebraic manipulation program.

## List of Acronyms, Abbreviations and Symbols

**Models**
DLG: Doniach lattice gas model
BEG: Blume−Emery−Griffiths model
**Approximations**
MFA: mean-field approximation
BPA: Bethe−Peierls approximation
**Lipid Single-Site States**
o: ordered (gel-like)
d: disordered (liquid-like)
w: vacant (water-filled)
**Monolayer Thermodynamic Phases**
G: gas
LE: liquid expanded
LE1: liquid expanded 1
LE2: liquid expanded 2
LC: liquid condensed
SC: solid crystalline
Stg: staggered
**Transition Lines**
L1: first-order line (dashed)
L2: second-order line (solid)
CL: critical line (solid)
**Multicritical Points**
CP: critical point (●)
CEP: critical end point (■)
TCP: tricritical point (◆)
TP: triple point (▲)
**Sponsors**
CAPES: Coordination for the Improvement of Higher Education Personnel
CNPq: National Council for Scientific and Technological Development
FAPESP: São Paulo Research Foundation
INCT-FCx: the National Institute of Science and Technology Complex Fluids
**Other Abbreviations and Acronyms**
eq/eqs: equation/equations
ref/refs: reference/references
e.g.: *exempli gratia* = for example
i.e.: *id est* = that is
mN/m: millinewton/meter
DMPC: 1,2-dimyristoyl-*sn*-glycero-3-phosphocholine
